# Supplementary material for: Epigenetic Activation of TUSC3 Sensitizes Glioblastoma to Temozolomide Independent of MGMT Promoter Methylation Status
Source: Int J Mol Sci. 2023 Oct 14;24(20):15179. doi: 10.3390/ijms242015179 (PMC10606804; doi:10.3390/ijms242015179)
Supplement: Supplementary file 1 [file ijms-24-15179-s001.zip › Supplemental Figures_Revision_Final.pdf]

Figure S1

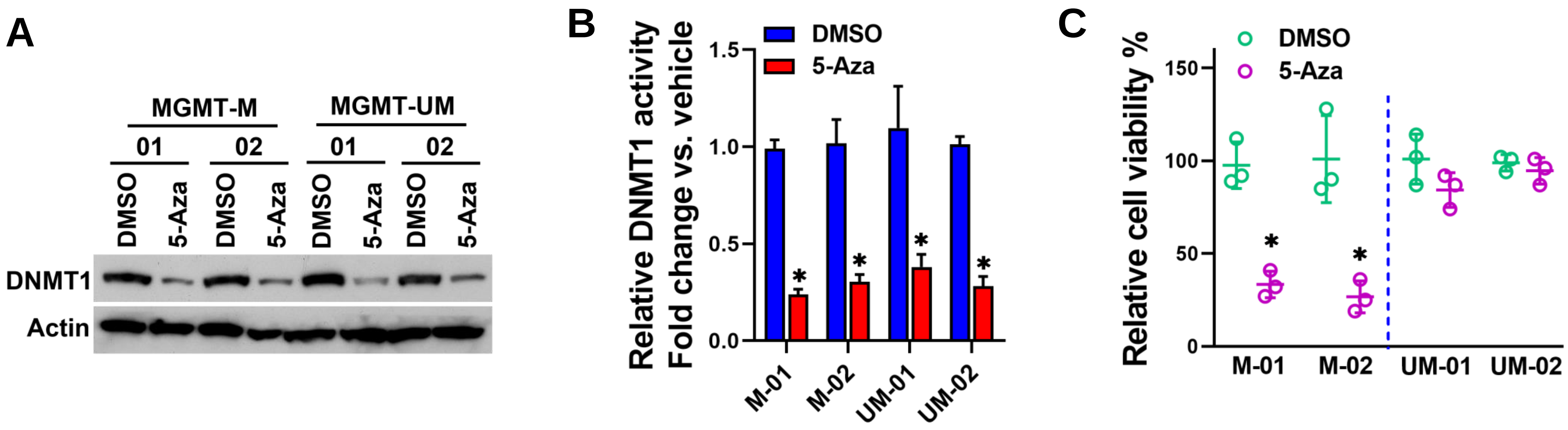

Figure S1 (A) Immunoblotting of DNMT1 protein in MGMT-M and MGMT-UM GSCs. β-actin was used as loading control. (B) DNMT1 activity measurement in MGMT-M and MGMT-UM GSCs when treated with DMSO control and 5-Aza for 72 hours. (C) Relative cell viabilities of GSCs under 5-Aza or DMSO treatment in combination with TMZ for 72 hours. Data are presented as mean ± SD. n=3; *, $p<0.05$.

Figure S2

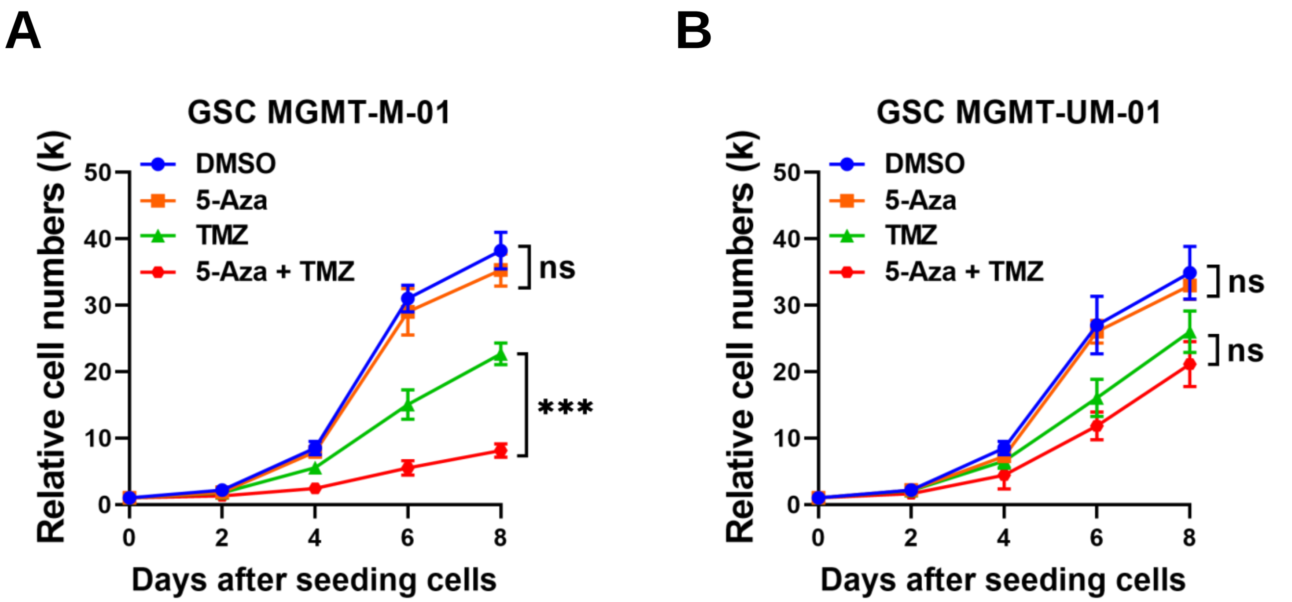

Figure S2 Cell growth curves of MGMT-M (A) and MGMT-UM (B) GSCs, under 4 groups treatment indicated respectively. Data are presented as mean ± SD. n=3; ***, p<0.001; ns, no significance.

Figure S3

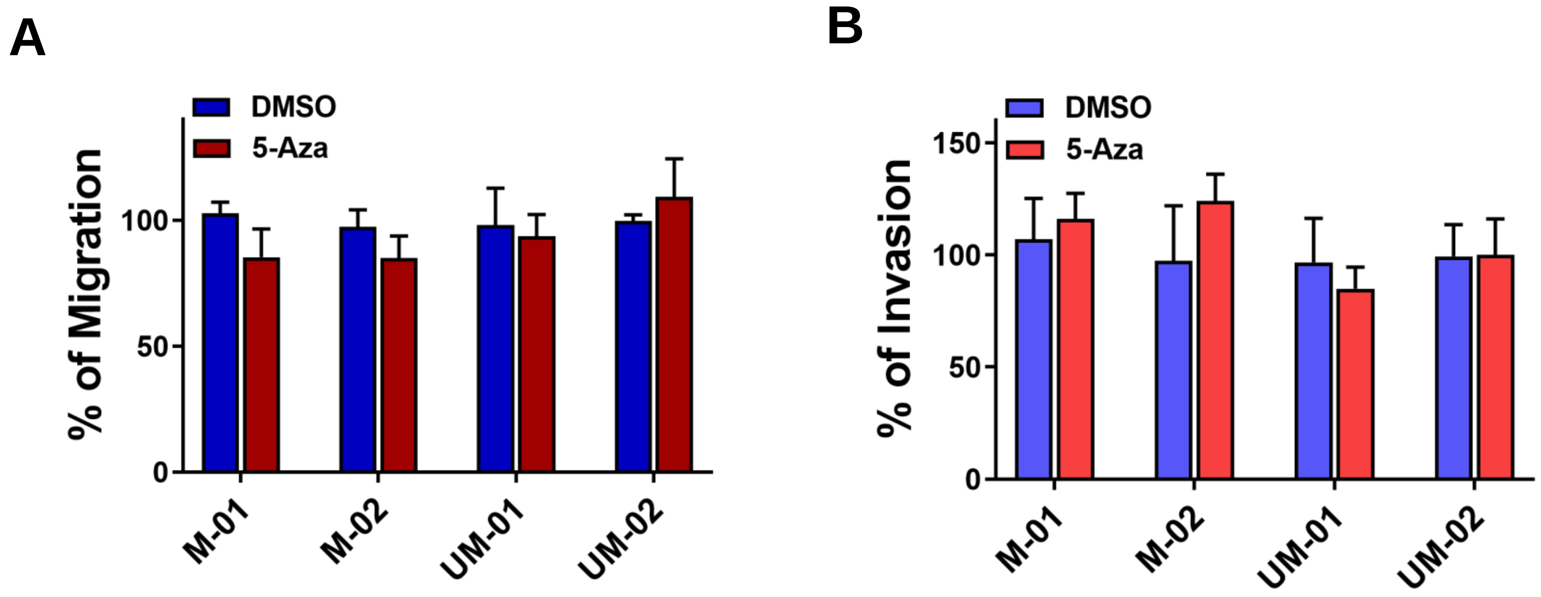

Figure S3 (A-B) Trans-well migration and invasion assay of MGMT-M and MGMT-UM GSCs, DMSO control versus 5-Aza treatment. DMSO treated cell numbers were normalized as 100%. Data are presented as mean ± SD. n=3.

Figure S4

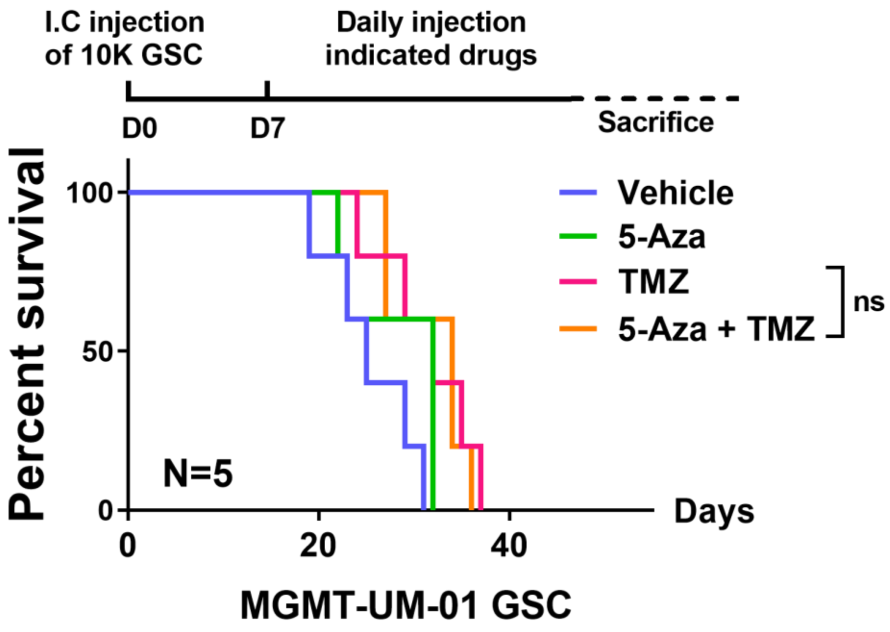

Figure S4 Kaplan-Meier survival curves of immunocompromised mice bearing MGMT-UM GSCs treated with Vehicle, 5-Aza only, TMZ only or 5-Aza plus TMZ. The p values were calculated by Mantel-Cox log-rank test. ns, no significance. I.C., intracranial.

Figure S5

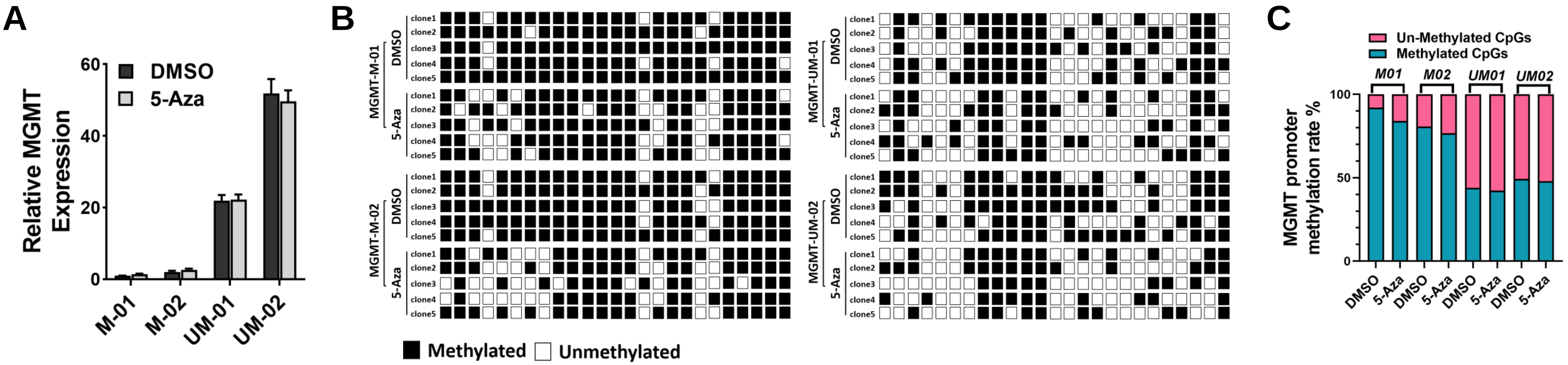

Figure S5 (A) MGMT mRNA expression examined by qPCR in MGMT-M and MGMT-UM GSCs treated with DMSO or 5-Aza (72 hours). Data are presented as mean ± SD. (B) Bisulfite sequencing was performed of human MGMT promoter in MGMT-M and MGMT-UM GSCs. Each square represents one CpG-dinucleotide. Black square indicates methylated CpG-dinucleotide and white square indicated unmethylated CpG-dinucleotide. (C) Quantification of indicated bisulfite sequencing result in (B).

Figure S6

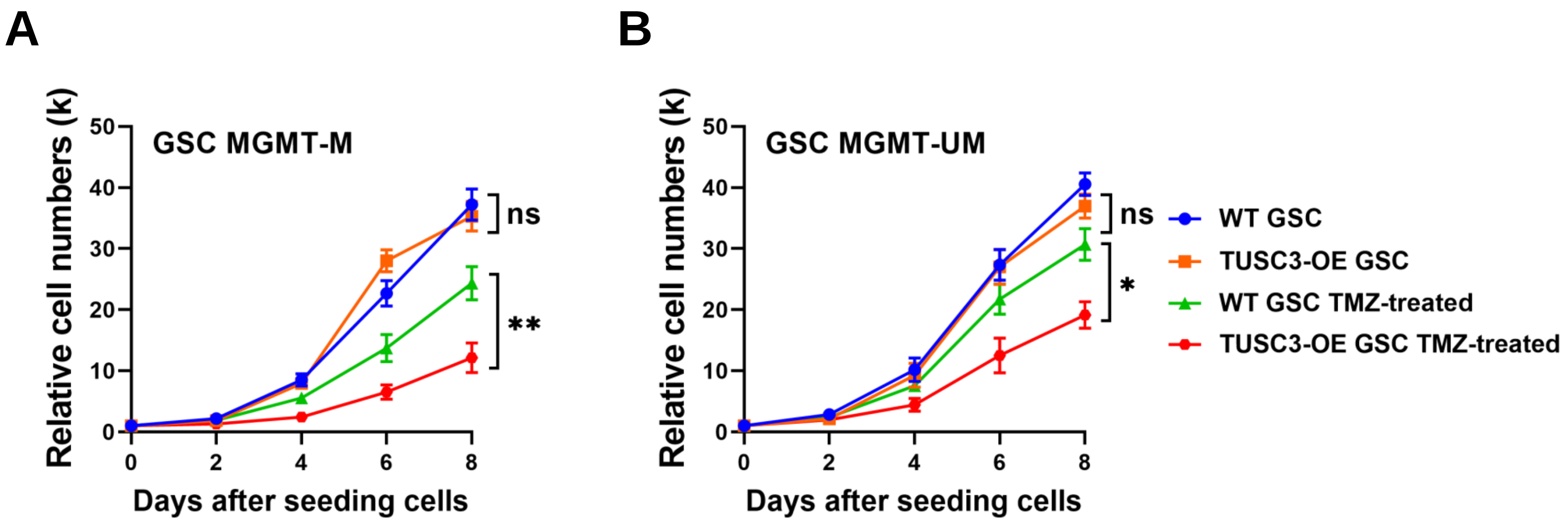

Figure S6 Cell growth curves of MGMT-M (A) and MGMT-UM (B) GSCs. 4 groups treatment indicated respectively including: wild-type GSC (DMSO treated), TUSC3-OE GSC (DMSO treated), wild-type GSC with TMZ treatment, and TUSC3-OE GSC with TMZ treatment. Data are presented as mean ± SD. n=3; *, $p<0.05$; **, $p<0.01$; ns, no significance.

Figure S7

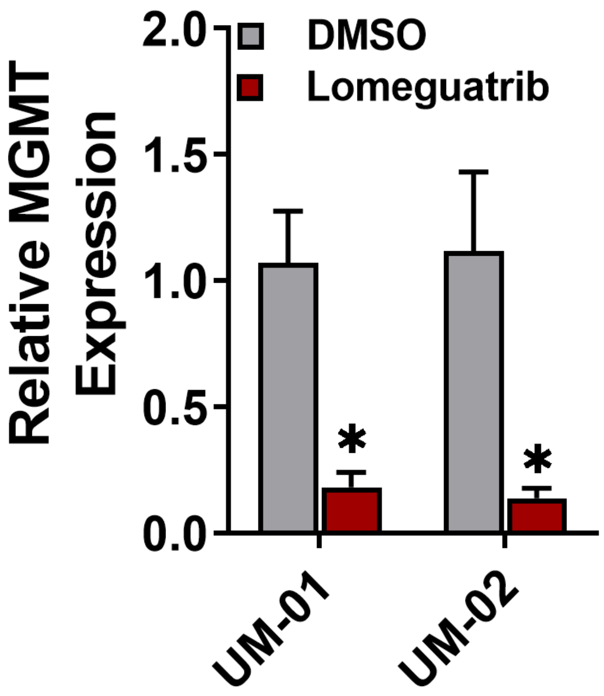

Figure S7 Inhibition efficiency of MGMT by Lomeguatrib examined by qPCR in MGMT-UM GSCs. Data are presented as mean ± SD. n=3; *, $p<0.05$.

Figure S8

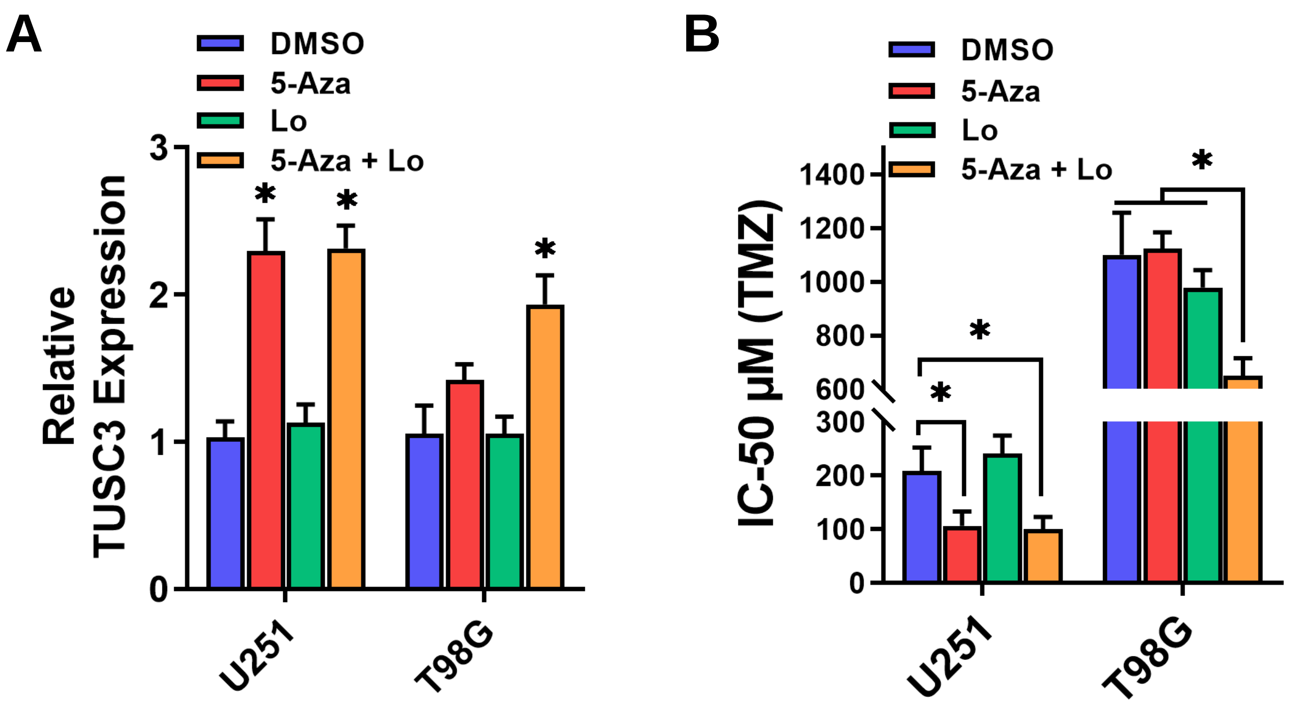

Figure S8 (A) mRNA expression of TUSC3 was measured by qPCR in U251 and T98G GBM cells with indicated single or combination treatment. (B) TMZ IC-50 alteration during indicated treatment (4 treatment groups) in U251 and T98G GBM cells. All cells were treated for 72 hours. 5-Aza, 20 µM; Lomeguatrib, 20 µM. Lo, Lomeguatrib. Data are presented as mean ± SD. n=3; *, $p<0.05$.

Figure S9

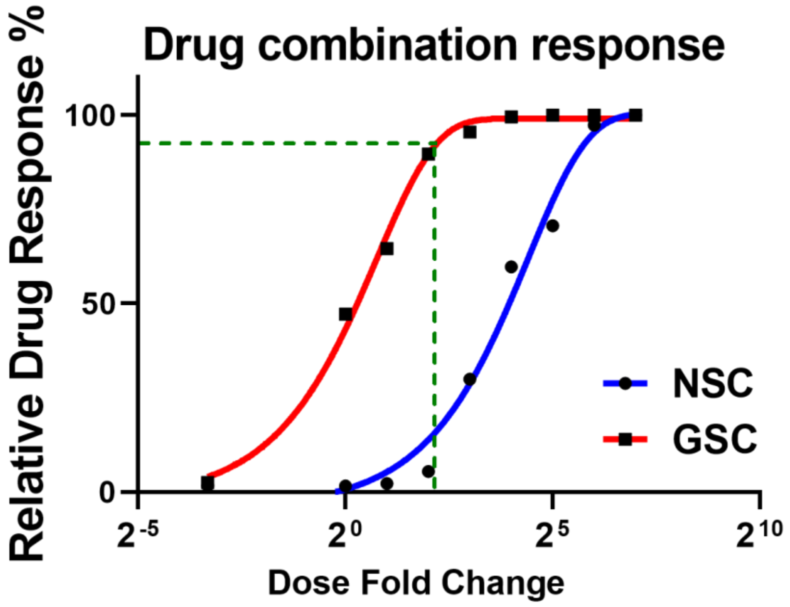

Figure S9 Dose-response curve of 5-Aza, TMZ and Lomeguatrib three-drug combination in MGMT-UM GSC and NSC for 72 hours with a serial of 2-fold dose escalation. Initial doses: 5-Aza, 5 µM; TMZ, 50 µM; Lomeguatrib, 5 µM.
